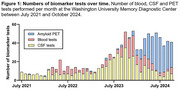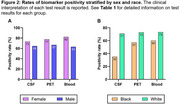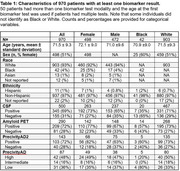# Rates of Alzheimer disease biomarker positivity stratified by by sex and race in a specialty memory clinic

**DOI:** 10.1002/alz70856_105627

**Published:** 2026-01-08

**Authors:** Anna Hofmann, Madeline Paczynski, Zachary J Posey, Melissa Aldinger, Tammie L.S. Benzinger, John C. Morris, B. Joy Snider, Suzanne E. Schindler

**Affiliations:** ^1^ Knight Alzheimer Disease Research Center, St. Louis, MO, USA; ^2^ Washington University School of Medicine, St. Louis, MO, USA; ^3^ Knight Alzheimer Disease Research Center, Saint Louis, MO, USA; ^4^ Washington University School of Medicine, Saint Louis, MO, USA; ^5^ Washington University, Saint Louis, MO, USA; ^6^ Department of Radiology, Washington University School of Medicine, St. Louis, MO, USA; ^7^ Washington University School of Medicine in St. Louis, St. Louis, MO, USA; ^8^ Mallinckrodt Institute of Radiology, Washington University School of Medicine, St. Louis, MO, USA; ^9^ Knight Alzheimer Disease Research Center, Washington University School of Medicine, St. Louis, MO, USA; ^10^ Department of Neurology, Washington University School of Medicine, St. Louis, MO, USA; ^11^ Washington University in St. Louis, St. Louis, MO, USA

## Abstract

**Background:**

The Washington University Memory Diagnostic Center (MDC) in St. Louis, Missouri, annually sees approximately 4,000 patients with memory and thinking concerns and uses multiple biomarker modalities to assess for AD pathology, including to establish eligibility for anti‐amyloid treatments. We assessed the use of different biomarker modalities over time and the rates of biomarker positivity by sex and racial group.

**Method:**

Biomarker positivity was based on the clinical interpretation of the Elecsys *p*‐tau181/Aβ42 CSF test (Roche), the PrecivityAD and PrecivityAD2 blood tests (C2N Diagnostics), and Florbetaben or Florbetapir amyloid PET scans. Clinical and demographic data were retrospectively collected from electronic health records.

**Result:**

A total of 970 patients were included who underwent at least one AD biomarker test between June 2021 and December 2024 at the MDC and/or anti‐amyloid treatment initiation. The average patient age was 71.5 ± 9.3 years (mean ± standard deviation), 51% were female, and 93% were White. The numbers of tests performed were as follows: 500 CSF tests, 290 amyloid PET scans, 143 PrecivityAD2 blood tests, and 87 PrecivityAD blood tests (Table 1). 50 patients underwent testing with more than one biomarker modality. The monthly volume of biomarker testing increased from ∼10 patients/month from July 2021‐July 2022 to ∼40 patients/month from July 2023‐July 2024 (Figure 1). Females were more likely than males to be biomarker positive: for CSF tests, 73% females and 65% males were positive, *p* = 0.041; for amyloid PET, 77% females and 67% males were positive, *p* = 0.045; for the PrecivityAD2 blood test, 82% females and 63% males were positive, *p* = 0.010 (Figure 2a). Only 42 individuals undergoing biomarker testing were Black (4%). White individuals were more likely than Black individuals to be biomarker positive on CSF tests (71% White and 35% Black were positive, *p* = 0.0018); few Black individuals underwent amyloid PET or blood tests (Figure 2b).

**Conclusion:**

The MDC used a combination of CSF tests, blood tests, and amyloid PET scans to scale up AD biomarker testing over the past three years. Rates of AD biomarker positivity were approximately 70% across all three modalities, with females and White individuals having higher rates of positivity.